# Appendicite sur appendice diverticulaire

**DOI:** 10.11604/pamj.2021.40.253.32464

**Published:** 2021-12-21

**Authors:** Mohamed Hedfi, Hakim Zenaidi

**Affiliations:** 1 Service de Chirurgie Générale, Hôpital Régional de Zaghouan, Zaghouan, Tunisie

**Keywords:** Appendice, diverticule, chirurgie, Appendix, diverticulum, surgery

## Abstract

Diverticulum of the appendix is defined as the existence of diverticulum (s) arising from the cecal appendix resulting in polymorphic clinical manifestations characterized by infection of the appendix and the periappendicular region. We here report the case of a 28-year-old female patient without a significant medical history, presenting to the Emergency Department with fever, right iliac fossa pain associated with vomiting. Clinical examination showed a temperature of 38.5°C, clear susceptibility of the right iliac fossa with positive Blumberg sign and right rectal pain on touch. Differential diagnoses of urinary tract infection were excluded based on negative cytobacteriological examination of the urine. Other diagnoses, such as Crohn´s disease and colon cancers, were also excluded based on patient´s history and context. The diagnosis of acute appendicitis was made and the patient underwent coelioscopy that revealed internal laterocecal appendix with phlegmon and two diverticules at the tip and body of the appendix (A: a coelioscopic view of the appendix phlegmon, seat of the diverticulum). Appendicectomy was performed (B: macroscopic view of the appendix showing the diverticula) without incident. Histological examination showed acute appendicitis with uncomplicated diverticulitis. The postoperative course was uneventful.

## Image en médecine

La diverticulose appendiculaire se définit comme l´existence de diverticule(s) sur l´appendice cæcal, réalisant des tableaux cliniques polymorphes avec infection de l´appendice et de la région péri-appendiculaire. On présente le cas d´une jeune patiente de 28 ans sans antécédents pathologiques notables consultant en urgence pour syndrome douloureux et fébrile de la fosse iliaque droite associé à des vomissements. L´examen de l´abdomen trouve une température à 38,5°C, une nette sensibilité de la fosse iliaque droite avec un signe de Blumberg positif et un toucher rectal douloureux à droite. Les diagnostics différentiels en particulier avec une infection urinaire ont été éliminés devant la négativité de l´examen cytobactériologiques des urines. D´autres diagnostics tels que la maladie de Crohn et les tumeurs coliques étaient éliminés du fait de l´anamnèse et du contexte. Le diagnostic d´appendicite aiguë a été porté, le patient a été opéré par voie coelioscopique permettant de découvrir un appendice latérocecal interne phlegmoneux avec présence de deux diverticules au niveau de la pointe et du corps de l´appendice (A: vue peropératoire coelioscopique de l´appendice phlegmoneux siège de diverticules). Il a été réalisé une appendicectomie (B: vue macroscopique de l´appendice montrant les diverticules) sans incidents et l´examen histologique avait conclu à une appendicite aiguë avec diverticules appendiculaires non compliqués. Les suites opératoires étaient simples.

**Figure 1:**
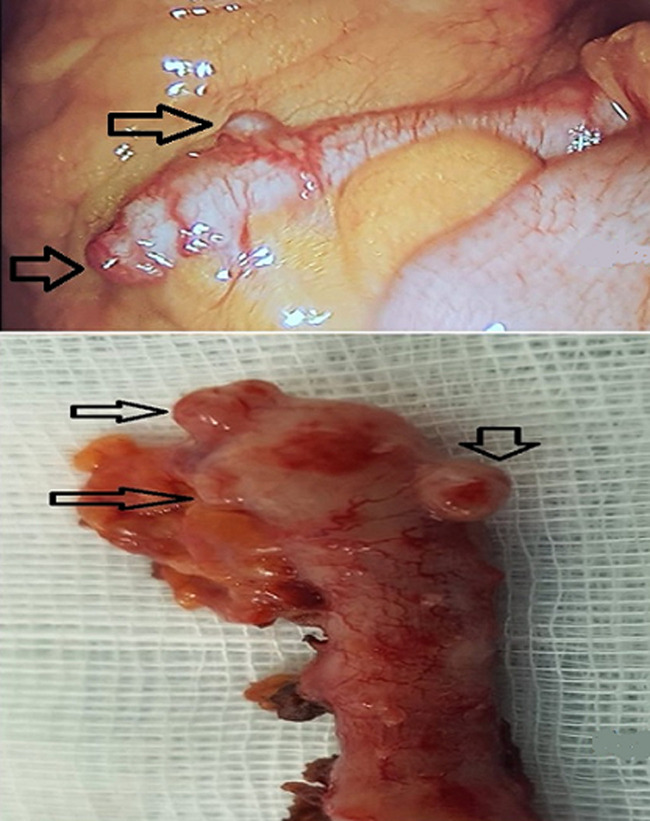
A) vue peropératoire coelioscopique de l´appendice phlegmoneux siège de diverticules; B) vue macroscopique de l´appendice montrant les diverticules

